# Impact of Gastropexy/Omentopexy on Gastrointestinal Symptoms after Laparoscopic Sleeve Gastrectomy

**DOI:** 10.1007/s11695-021-05806-y

**Published:** 2021-12-06

**Authors:** Hady Saleh Abou-Ashour

**Affiliations:** General Surgery, Menoufia Faculty of Medicine, General Surgery Department, Menoufia University Hospital, Shebeen El-Kom, Egypt

**Keywords:** Laparoscopic sleeve gastrectomy, Omentopexy, Gastropexy, Gastroesophageal reflux after sleeve gastrectomy

## Abstract

**Background:**

Laparoscopic sleeve gastrectomy (LSG) has become a single-step operation for the management of severe obesity. A statistically significant number of participants who undergo this procedure experience nausea, vomiting, and reflux symptoms early after the operation. The objectives of this study were to measure the positive or negative effect of gastropexy on reducing distressing postoperative LSG-related gastrointestinal symptoms.

**Patients and Methods:**

This was a comparative randomized study conducted from January 2018 to January 2021. The study was carried out in the general surgery department at Menoufia University Hospital, Menoufia Faculty of Medicine in Egypt. Two hundred participants were included randomly during this trial. The participants were divided into two groups, with 100 patients in each group. Patients in group A underwent gastropexy, and patients in group B underwent LSG without gastropexy.

**Results:**

There was no significant difference between the groups in age or sex (*p* > 0.05). There was no significant difference in the length of hospital stay (*p* > 0.05). There was a significant difference between the two groups regarding nausea, vomiting, reflux symptoms, and the amount and frequency of antiemetics used (*p* < 0.001). There was also a significant difference in hospital readmissions (*p* < 0.05) and in clinic visits during the postoperative period.

**Conclusions:**

Patients who underwent gastropexy showed a significant reduction in antiemetic consumption and a significantly lower incidence of postoperative nausea, vomiting, gastroesophageal reflux disease symptoms and gastric torsion than those who did not undergo gastropexy.

**Graphical abstract:**

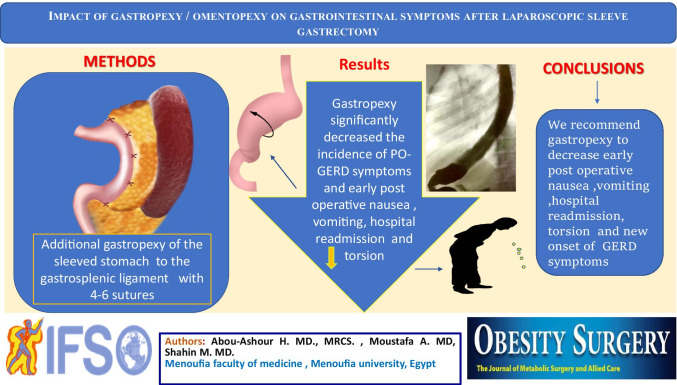

## Introduction

In 1993, Marceau described sleeve gastrectomy, which aims to promote weight loss, as a component of biliopancreatic diversion [1]. Reduction of the gastric volume reduces the volume of food that can be consumed, with a subsequent reduction in food utilization [2]. Additionally, the decreased volume of the new stomach results in earlier distention, resulting in the stimulation of stretch receptors within the wall of the stomach [3]. This leads to lower levels of ghrelin due to reductions in the gastric fundus and ghrelin production, likely causing greater satiety [4].

Laparoscopic sleeve gastrectomy (LSG) has become one of the most commonly performed bariatric procedures and has been accepted by the American Society for Metabolic and Bariatric Surgery as a first-stage operation for morbidly obese patients [5, 6].

Patients may complain of significant nausea within the follow-up period, which leads to additional clinic visits, telephone encounters, and readmissions [7]. Excitation of old or new gastroesophageal reflux and anorexia are the most common complications after LSG. These complications may have considerable effects on quality of life and may require conversion to another operation, such as Roux-en-Y gastric bypass (RYGB) [8]. Some trials have reported that loss of gastric fixation could lead to improper positioning of the sleeved stomach, causing permanent gastroesophageal reflux and anorexia [9]. Moreover, volvulus has been reported after LSG [10].

Patients treated with LSG report a group of distressing gastrointestinal (GI) symptoms that can lengthen their hospital stay, reduce their quality of life and place an extra burden on the hospital team. Distressing postoperative symptoms and frequent clinic visits badly affect the reputation of bariatric surgery [11]. LSG has been associated with improvement in and/or excitation of gastroesophageal reflux disease (GERD) in obese patients [12]. The incidence of GERD is 22% after LSG, although this percentage decreases after several years [13]. Again, Carter and colleagues reported that 47% of patients who underwent LSG had permanent GERD symptoms [14].

Bauman et al. [15] analyzed the stomach anatomy after LSG by examining thirty-two multislice computed tomography datasets from twenty-seven participants. Forty percent of the participants had intrathoracic migration of the staple line, causing continuous nausea during the postoperative period.

Abe et al. reported that omentopexy maintains the stomach intra-abdominally and prevents intrathoracic migration. Additionally, the loss of appropriate positioning of the stomach can contribute to anorexia [16]. In the present study, our hypothesis was that gastropexy could decrease the incidence of common GI symptoms, such as nausea, vomiting, and reflux.

In the present study, we compared two groups to investigate any reduction in potential complications that have been associated with a lack of fixation of the new stomach.

## Patients and Methodology

This was a comparative randomized study conducted from January 2018 to January 2021 in the General Surgery Department at Menoufia University Hospital in Egypt. Two hundred patients were decided to be included in the study. Then patients were recruited from the hospital outpatient clinic. The closed envelope technique has been used as a method of randomization. For building two equal groups, there were one hundred envelopes containing LSG with gastropexy and another 100 envelopes containing LSG without gastropexy. Then 100 larger envelopes were prepared. Each envelope contained 2 different envelopes from the above techniques. Each participant was offered a large envelope to choose one smaller envelope as a double-blind technique (neither the doctors nor the participants knew the chosen operation). According to their random choices, participants were then divided into two different equal groups- automatically, 100 each.

Group A (100 participants) underwent LSG with gastropexy, while patients in group B (100 participants) underwent LSG without gastropexy.

- Preoperative measures: All patients underwent a complete history taking, complete clinical examination, body mass index (BMI) measurement, upper GI endoscopy, and routine laboratory investigations.
- Radiographic barium study was reserved for patients with persistent GERD symptoms or suspected torsion after surgery.

The primary study endpoint is the impact of gastropexy or no gastropexy on the following:

1. Post-operative nausea and vomiting within the 1^st^ months after surgery.
2. Post-operative reflux symptoms along the 1^st^ postoperative year.

The secondary study endpoint is the impact of gastropexy or no gastropexy on the following:

1. The hospital stay and hospital readmission
2. Symptom-related post postoperative clinical visits during the 1^st^ month.
3. The usage of antiemetics. (More than 16 mg/day of Ondansetron and/or 10 mg Metoclopramide/day) during the 1^st^ postoperative month.
4. All data were collected prospectively.
5. The retrospective results of valuable significance were also reported.

Inclusion criteria were as follows:

1. BMI ranging from 35 to 65 kg/m^2^
2. Age ranging from 18 to 65 years

Exclusion criteria were as follows:

1. Pregnancy
2. Severe psychiatric disease
3. Hormonal abnormalities, e.g., Cushing disease or hypothyroidism
4. Reflux symptoms or hiatal hernia
5. GI diseases, such as Crohn’s disease or GI anomalies
6. Inability to undergo anesthesia

The mean clinical score of GER reduced from 10.7 in the preoperative period to 0.7 in the postoperative period (Filho et al., 2018). So, the sample size is 200 (100 in each group). The sample was calculated using an open Epi program with a confidence level of 95% and power of 80%.

Conventional laparoscopic gastrectomy was performed using a 36-Fr calibration tube in all patients. Patients in group A also underwent gastric fixation with 4–6 interrupted sutures starting from the proximal end of the stomach down to the distal end. The 1^st^ suture site was between the proximal end of the stomach and the upper end of the gastrosplenic ligament. The distance between gastropexy sutures was nearly equal to the length of each fired staple. Most of the participants had 4–5 sutures and more sutures were reserved for those with long stomachs. Ex: The length of five fires required 6 sutures.

**Fig. 1:**
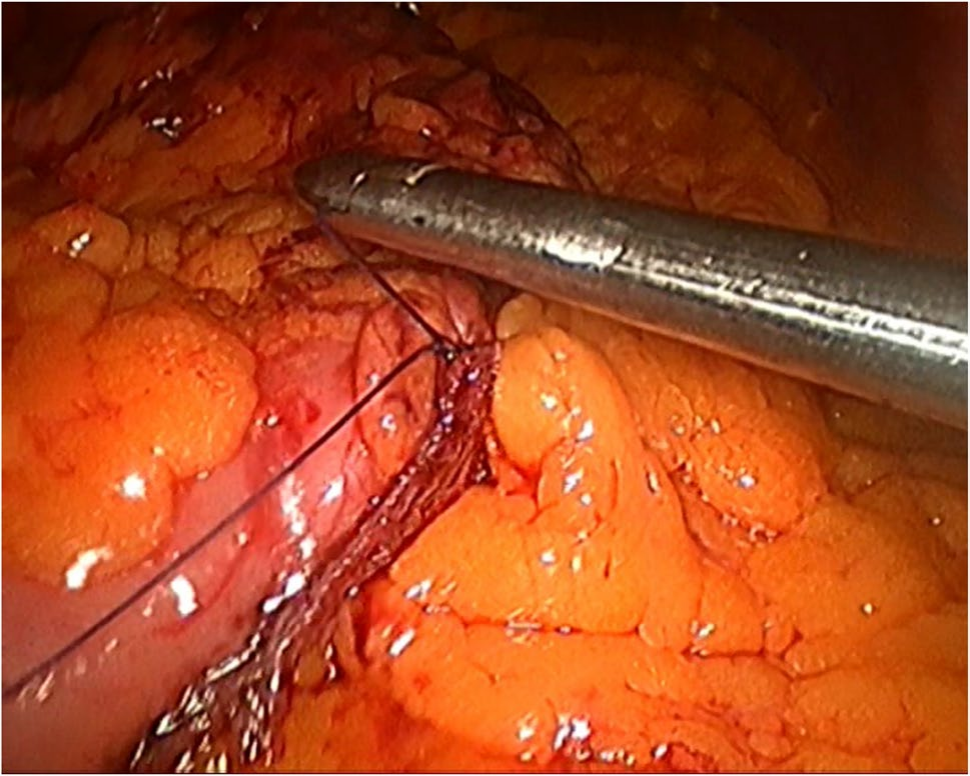
Gastropexy suturing

**Fig. 2:**
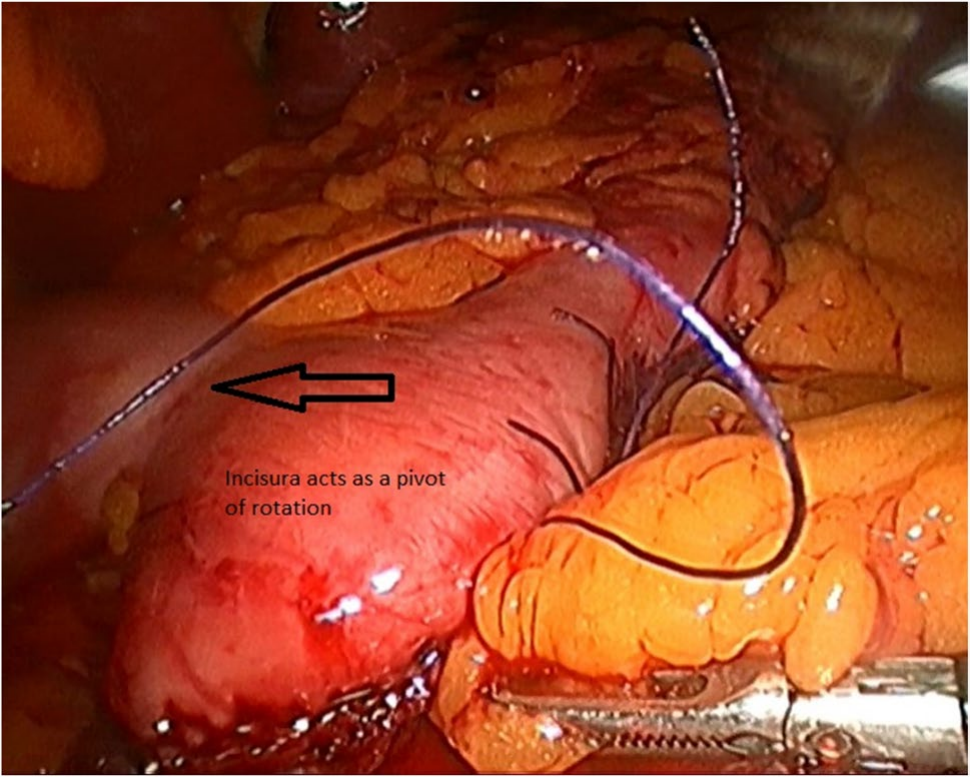
Incisura acts as a pivot of rotation

**Fig. 3:**
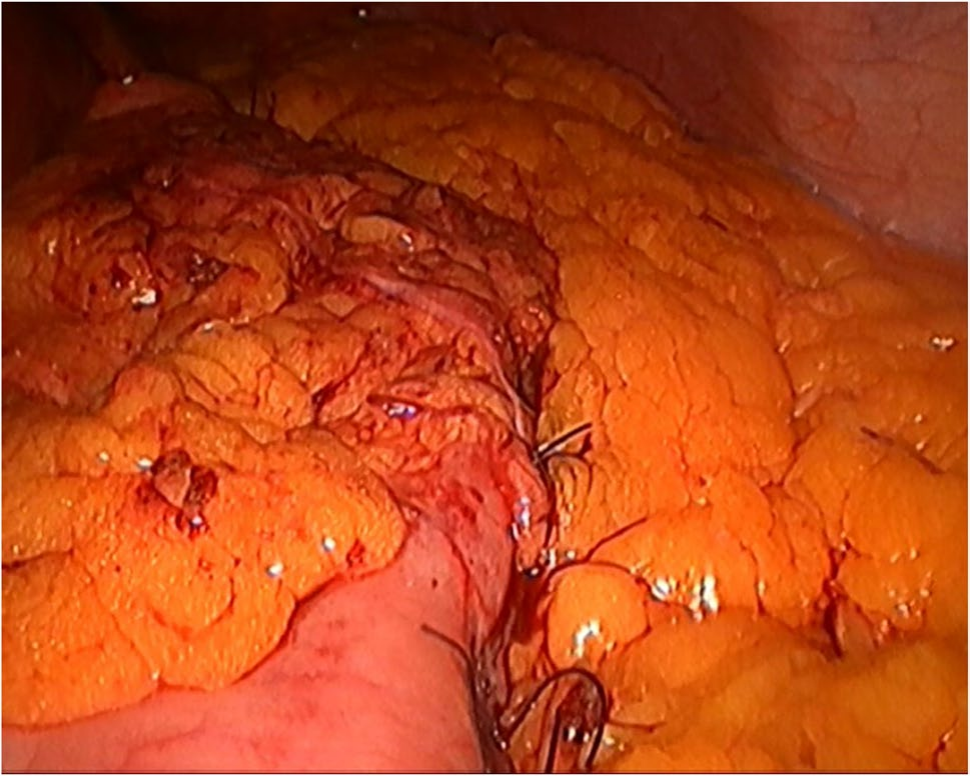
Gastropexy is completed

Fixation was performed by simple suturing with Prolene, Monocryl, or Vicryl (3:0 round needle). The steps are shown in Figs. 1, 2, and 3.

Conventional routine postoperative management was performed, and a clear liquid diet was started on the second day after surgery.

The patients were given thromboembolic prophylaxis in the form of low-molecular-weight heparin for 15 days. Oral antibiotics were prescribed for 1 week (amoxicillin + clavulanic acid [Augmentin], 1 gm/12 h).

A proton pump inhibitor (PPI) was prescribed for 3 months. The dose and frequency of antiemetic consumption (metoclopramide and Zofran) were recorded.

In the present study, we compared two groups to investigate any reduction in potential complications that have been associated with a lack of fixation of the new stomach.

The patients in each group were monitored to determine the following:

- Number of postoperative complaint calls
- Number of vomiting attacks
- Number of nausea instances reported
- Number of hospital readmissions
- Incidence of reflux symptoms
- Incidence of any postoperative complications

All of the above items were statistically analyzed.

- The patients were followed up for 1 year.

## Statistical Analysis

For data collection, we used a Microsoft Access database (Office 2000). Statistical analysis was performed using Epi Info 7.0 software. A two-sided *p* value of less than 0.05 was considered significant. Bivariate analyses were performed using the *χ*2 test or Fisher’s exact test for categorical data and the independent-sample *t* test for continuous data. All results are presented as the mean and standard deviation (± SD) unless otherwise stated.

## Results

The mean age in groups A and B was 36 ± 1 (range, 24–61) and 35 ± 12 (range, 19–65) years, respectively. The mean BMI in groups A and B was 44 ± 6 and 45 ± 3 kg/m^2^ (range, 36–55), respectively.

The mean operative duration was 63 ± 9.5 min in group A and 58 ± 10 min in group B (*p* > 0.05).

The mean length of hospital stay was 28 ± 9 h and 35 ± 8 h in groups A and B, respectively (*p* = 0.07).

Postoperative leakage did not occur in any patient in either group.

Nausea disappeared 48 h after surgery in most patients in the current study. Prolonged nausea occurred in 6 patients in group A and 15 patients in group B during the 1^st^ month (*p* < 0.001). In group A, nausea was mild but more severe in group B. Severe prolonged nausea was associated with pain which required pain medication. Patients who had severe nausea in group B with persistent vomiting and pain required hospitalization and they were managed by conservative treatment.

In group A, postoperative vomiting occurred in 8 patients during the 1^st^ month; in group B, vomiting occurred in fourteen patients, and two of whom were readmitted. Both of them responded to conservative treatment.

There was a statistically significant difference between the groups regarding postoperative vomiting (*p* < 0.001) and hospital readmission (*p* < 0.05).

In group B, one patient experienced prolonged dyspepsia, abdominal discomfort, and recurrent and persistent vomiting. He was diagnosed with gastric torsion 4 months after surgery. This patient underwent laparoscopic torsion reduction and gastropexy. In the current study, no patients had gastric torsion in group A (*p* < 0.05).

No cases of postoperative bleeding were reported in either group.

Regarding postoperative reflux symptoms, patients in group A reported a much lower incidence of reflux symptoms (6%) than those in group B (18%) (*p* < 0.001).

An important retrospective item was noticed. We contacted all of the participants and discussed with them the amount and duration of PPIs usage. We found that both the dose and duration were lesser in group A than in group B (*p* < 0.001) (Table 3).

PPIs, patients in group B reported prolonged use of these medications to control reflux symptoms (> 3 months).

**Fig. 4:**
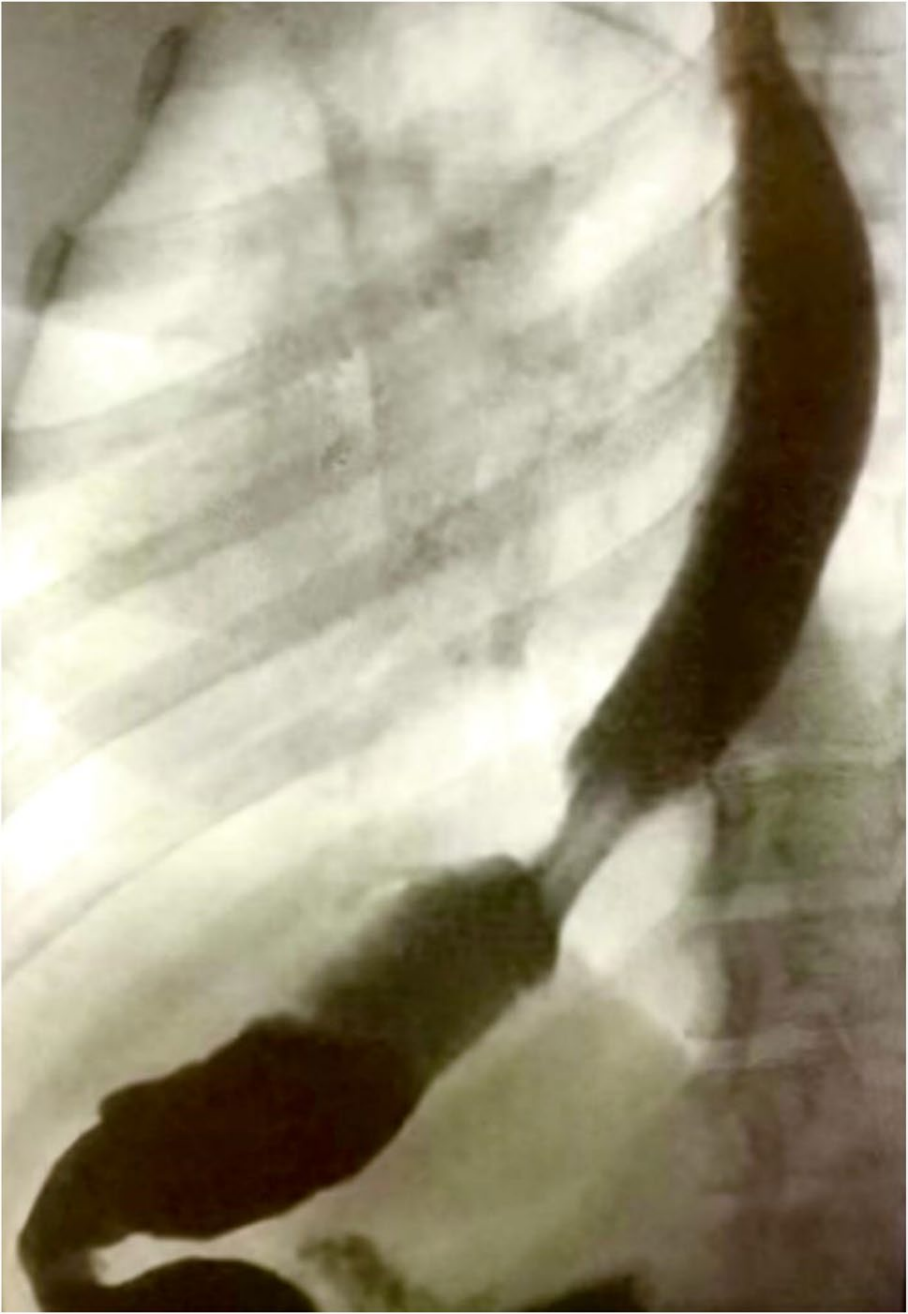
Complete incompetence of the GEJ 9 months after surgery

Additionally, two patients suffered severe gastroesophageal junction (GEJ) incompetence (Fig. 4).

Results are shown in Tables 1, 2, and 3.

**Table 1:** Patient demographics

	Group A		Group B		*p* value
Mean age	36 ± 14 years		35 ± 12 years		0.4
Sex	Males	Females	Males	Females	0.5
	37	63	41	59	
Mean BMI	44 ± 6		45 ± 3		0.3

**Table 2:** Operative duration and length of hospital stay

	Group A	Group B	*p* value
Mean operative duration	63 ± 9.5 min	58 ± 10 min	0.09
Mean length of hospital stay	28 ± 9 h	35 ± 8 h	0.07
Number of gastropexy sutures	4 ± 1 range (4–6)	–

**Table 3:** Postoperative complications

	Group A	%	Group B	%	*p* value
PO nausea	6	6%	15	18%	*p* < 0.001
PO vomiting	8	8%	14	14%	*p* < 0.001
PO bleeding	0	0%	0	0%	-
Reflux symptoms in the 1^st^ year after surgery	6	6%	18	18%	*p* < 0.001
Gastric torsion	0	0%	1	1%	*p* < 0.05
Leakage	0	0%	0	0%	*p* > 0.05
Hospital readmission in 30 days	0	0%	2	2%	*p* < 0.05
Reoperation for complications	0	0%	1	1%	*p* > 0.05
PO antiemetic use					
Metoclopramide > (10 mg/day)	6	6%	14	14%	*p* < 0.001
Ondansetron > 16 mg/day	2	2%	13	13%	*p* < 0.001
Prolonged PPI use > 3 months)	8	8%	23	23%	*p* < 0.001
Severe GEJ incompetence	0	0%	2	2%	*p* < 0.05
Calls to hospital/surgeon during the 1^st^ PO week > 6 calls	9	9%	16	16%	*p* < 0.001
Excessive clinical visits (> 3) in the 1^st^ PO month	3	3%	9	9%	*p* < 0.05

## Discussion

LSG remains a good surgical option for severe obesity [17]. At present, the number of patients who undergo such surgery is high. Mathus-Vliegen reported that the loss of gastric fixation along its previous natural axis along the greater curve could also be an explanation for early postoperative nausea, retching, vomiting, and reflux symptoms [18].

Regarding GERD, LSG is associated with the improvement and/or excitation of GERD in obesity [19, 20]. The incidence is 22% early after the operation, although this percentage is reduced after several years [21]. The assumed mechanism for the increase in the incidence of GERD symptoms is the anatomy of the angle of His and sling fibers, with subsequent impairment of the lower esophageal sphincter [22]. Many patients improve with nonsurgical treatment; others are very resistant to high-dose medical treatment and will require conversion from LSG to RYGB [23].

Mokhtar et al. reported that vomiting was also a significant symptom after LSG. Vomiting occurred in 20% of patients and continued to be a troublesome symptom throughout the follow-up period; additionally, it was almost always associated with GERD. Vomiting was mildly responsive to repeated PPI therapy and was associated with incompetent cardia during endoscopy in 66.7% of the participants (*p* = 0.029) [24]. Altieri et al. reported that dysphagia also developed de novo in 13.3% of participants following LSG along with dyspeptic symptoms [7].

Bredenoord et al. believed that the anti-reflux barrier was located at the junction with the stomach and consisted of the lower esophageal sphincter and the crus of the diaphragm [25]. Kahrilas et al. reported a reduction in acidic chyme, a type of unbuffered, highly acidic digestive fluid, at the GEJ after eating. The presence of acidic fluid in a supradiaphragmatic location is a strong promoter of GERD [26]. There has been much debate regarding the optimal bougie size. To date, there is no consensus on the optimal bougie size in LSG. In the present study, we used a 36-Fr bougie in all participants to fix this factor. Previous research has shown no significant difference in the percent of excess weight loss with the use of smaller bougies; however, the use of smaller bougies resulted in a higher incidence of dyspepsia, anorexia, dehydration, and leakage [27–30].

In the present study, GERD symptoms were present in 18% of patients who did not undergo gastropexy, while the incidence of GERD symptoms was much lower in those who underwent gastropexy, at 6%. Gastroesophageal junction incompetence was diagnosed by reflux symptoms and radiography using barium swallow. Two participants (2%) in group B experienced severe and continuous reflux symptoms 9 months after surgery and significant GEJ incompetence (*p* < 0.05), as shown in Fig. 4, necessitating RYGB. Additionally, the low incidence of GERD after gastropexy highlights the importance of gastric fixation. A German cohort trial reported that the incidence of endoscopic GERD was 24.8% in patients [31], and another cohort study reported that the incidence of GERD was 31.4% [32]; the above results indirectly support the postulated benefits of gastropexy. In the present study, we used the term gastropexy rather than omentopexy, as our target was to fix the stomach, not the omentum. We used 4 to 6 sutures to fix the axis of the greater curvature, and there was no need to apply continuous sutures for the entire length of the greater curvature. Most of group A patients underwent gastropexy by 4 sutures but 5 and 6 sutures were reserved for those with long stomachs to avoid kink, to maintain sound fixation of the long greater curve, and to prevent internal hernia due to wide spacing. Fixation to the left crus of the diaphragm was tried but it caused tension to the upper part of the stomach and we found it more suitable to fix it to the upper part of the gastrosplenic ligament.

We also agree with Ponsky et al. and Poncet et al., who reported that it is important to maintain the GEJ at or below the level of the diaphragm to prevent intrathoracic sleeve migration. Gastropexy, with stitching of the gastrocolic and gastrosplenic ligaments (with the gastroepiploic arcade) to the staple line, may in theory address those factors while providing mechanical narrowing around the esophageal hiatus. To reduce recurrence after the laparoscopic management of hernias in the paraesophageal region, gastropexy is effective [33, 34].

A trial in Brazil [35] and a trial in the USA focused on anorexia during the early postoperative period [8]. These trials found a lower GERD score in those treated with gastropexy than in those treated without gastropexy.

Gawande et al. reported that recreating a normal anatomical position by gastropexy ameliorated anorexia and gastric torsion; the gastrohepatic, gastrocolic, and gastrosplenic ligaments were used to fix the stomach in place [36].

Functional gastric stenosis typically occurs at the level of the incisura angularis due to gastric torsion. The gastrophrenic, gastrosplenic, gastrocolic, and gastrohepatic ligaments that fix the posterior wall of the stomach are cut, and the new stomach becomes mobile. Gastric torsion or even volvulus may occur within a few days or months [37, 38]. Zigzagging of the staple line during resection of the stomach is another reason for gastric torsion or volvulus [39].

We noticed that gastropexy prevented the occurrence of gastric torsion in group A which had occurred in group B. Despite the statistically significant result in group B (*p* < 0.05), more studies are required with a larger number of patients to build a solid clinical relevance as regards gastric torsion.

Bredenoord et al. [25] reported that 40% of participants with the upward migration of the staple line inside the thorax experienced continuous nausea after surgery. Another study by Antonio et al. in 2019 concluded that omentopexy with LSG improved GERD in most cases, although it did not cause significant changes in the lower esophageal sphincter tone [40]. Again, our findings are in agreement with those reported by V. Vage et al. [41] in that gastropexy lowered the use of acid-reducing medications in patients who underwent gastropexy compared with patients who did not undergo gastropexy, as shown in Table 3. In the present study, we found that the patients in group A, who underwent gastropexy, had a lower incidence of nausea, vomiting, hospital readmission, reflux symptoms, and surgical reintervention and had no gastric torsion. Additionally, lower antiemetic use, fewer postoperative complaint calls, and a smoother postoperative recovery were observed in the gastropexy group. More studies by colleagues in bariatric surgery are needed to come to a final conclusion regarding the future of this technique and whether it should be standardized, omitted, or approved by bariatric surgeons.

## Conclusion

Patients who underwent gastropexy during LSG had a smoother postoperative course. Additionally, they showed a significant reduction in antiemetic usage and a significantly lower incidence of postoperative nausea, vomiting, GERD symptoms, and gastric torsion than those who did not undergo gastropexy.

### Study Limitations

The small sample size of the study, gastric torsion only occurred in one patient of group B and 99% didn’t have gastric torsion, more future studies are required from colleagues with a larger number of patients to build solid evidence and clinical relevance.

## Declarations

The current study was approved by the institutional ethical committee and all procedures performed in the study were in accordance with the ethical standards of the institutional and/or national research committee and with the 1964 Helsinki declaration and its later amendments or comparable ethical standards.
